# Rhizobacteria from ‘flowering desert’ events contribute to the mitigation of water scarcity stress during tomato seedling germination and growth

**DOI:** 10.1038/s41598-021-93303-8

**Published:** 2021-07-02

**Authors:** Marcia Astorga-Eló, Susett Gonzalez, Jacquelinne J. Acuña, Michael J. Sadowsky, Milko A. Jorquera

**Affiliations:** 1 Programa de Doctorado en Ciencias de Recursos Naturales, Universidad de La Frontera, Ave. Francisco Salazar, 01145 Temuco, Chile; 2 Laboratorio de Ecología Microbiana Aplicada (EMALAB), Departamento de Ciencias Química y Recursos Naturales, Universidad de La Frontera, Ave. Francisco Salazar, 01145 Temuco, Chile; 3 The Network for Extreme Environment Research (NEXER), Scientific and Biotechnological Bioresources Nucleus, Universidad de La Frontera, Ave. Francisco Salazar, 01145 Temuco, Chile; 4 Department of Soil, Water, and Climate, and Department of Plant and Microbial Biology, and BioTechnology Institute, University of Minnesota, 1479 Gortner Ave., St. Paul, MN 55108 USA

**Keywords:** Applied microbiology, Microbial ecology

## Abstract

Tomato (*Solanum lycopersicum* L.) is an important vegetable cultivated around the world. Under field conditions, tomato can be negatively affected by water scarcity in arid and semiarid regions. The application of native plant growth-promoting rhizobacteria (PGPR) isolated from arid environments has been proposed as an inoculant to mitigate abiotic stresses in plants. In this study, we evaluated rhizobacteria from *Cistanthe longiscapa* (*syn Calandrinia litoralis* and *Calandrinia longiscapa*), a representative native plant of flowering desert (FD) events (Atacama Desert, Chile), to determine their ability to reduce water scarcity stress on tomato seedlings. The isolated bacterial strains were characterized with respect to their PGPR traits, including P solubilization, 1-aminocyclopropane-1-carboxylate deaminase activity, and tryptophan-induced auxin and exopolysaccharide production. Three PGPR consortia were formulated with isolated *Bacillus* strains and then applied to tomato seeds, and then, the seedlings were exposed to different levels of water limitations. In general, tomato seeds and seedlings inoculated with the PGPR consortia presented significantly (*P* ≤ 0.05) greater plant growth (48 to 60 cm of height and 171 to 214 g of weight) and recovery rates (88 to 100%) compared with those without inoculation (37 to 51 cm of height; 146 to 197 g of fresh weight; 54 to 92% of recovery) after exposure to a lack of irrigation over different time intervals (24, 72 and 120 h) before transplantation. Our results revealed the effectiveness of the formulated PGPR consortia from FD to improve the performance of inoculated seeds and seedlings subjected to water scarcity; thus, the use of these consortia can represent an alternative approach for farmers facing drought events and water scarcity associated with climate change in semiarid and arid regions worldwide.

## Introduction

Currently, climate change is one of the main concerns for agriculture worldwide. Extensive drought periods and heat waves have been attributed to climate change, and they have led to significant losses in agriculture, especially in arid and semiarid regions globally^1,2^. In Chile, desertification processes represent a major risk and directly affect the vegetable production of the country because of the persistent drought conditions that have affected the country for the last 10 to 15 years^3^. To resolve this limitation, studies have proposed the search, selection and use of cultivars with a higher tolerance to stress because of water scarcity; however, this alternative is time consuming and costly to implement in many extensive agricultural areas in arid and semiarid regions affected by climate change^4,5^. In the same sense, farmers have been forced to maintain or improve the food supply in areas that have established irrigation systems to prevent water shortage stress in orchards; however, the use of low-quality water (e.g., rivers, estuaries and underground sources) has increased the salinity of soils, thereby affecting the growth and performance of plants^6^. Thus, efficient strategies to mitigate adverse climate events, such as droughts with consequent water scarcity and higher dehydration in plants, are highly required. Over the past decade, the relevance of microbiome-based science and plant growth-promoting rhizobacteria (PGPR) has been demonstrated by diverse studies in agriculturally relevant plants (such as crops, pastures, cereals, and fruit trees)^7,8^. The occurrence of PGPR has been reported not only in agriculturally relevant plants but also in native plants living in environments characterized by a permanent low availability of water and nutrients^9,10–12^. In these environments, native plants and their microbiota have coevolved under arid conditions; therefore, recent studies have proposed the use of PGPR from arid environments as inoculants to mitigate the damage of water limitation (drought) stress in plants. In this context, inoculation of wheat with consortia of PGPR isolated from the Atacama Desert (AD) improved plant growth under water shortage conditions^13^. While these results were encouraging, major efforts are still required to validate and implement this strategy at the commercial scale used in agriculture in arid and semiarid regions.

In Chile, the flowering desert (FD) phenomenon, also known as a blooming desert, was triggered by short and infrequent rainfall events, mostly located on the southern border of the Atacama Desert (from 30 to 29 °C S; from 68 to 70 °C W) during recent decades, which led to an increase in soil water available^14,15^. Subsequently, explosive growth of native plants was observed, with extremely high productivity that supported a rich but short-lived biotic assemblage^15,16^. Although a few studies have described the composition and functionality of rhizobacteria in plants during FD, most ecological implications remain unknown. Moreover, even fewer studies have examined AD as a source of PGPR that promotes the rapid growth and prevalence of plants in changing environments and their potential use for agriculture under water-limiting conditions. Thus, considering that agricultural production in templated and Mediterranean regions presenting water scarcity problems impacted by climate change-related events and the potential use of PGPR from arid environments to enhance vegetable production in these areas, we isolated and formulated rhizobacterial consortia from *Cistanthe longiscapa* (Barnéoud) Carolin ex M. A. Hershkovitz, a representative, native, and widely spreading plant during FD events, and then evaluated their effect on the seed germination, recovery and growth of tomato seedlings subjected to water shortage stress. Tomato was selected as a model plant because it is one of the main freshly consumed vegetables in Chile and its final production is very sensitive to environmental stresses at initial phenological stages^17^.

## Material and methods

### Sampling

**Figure 1:**
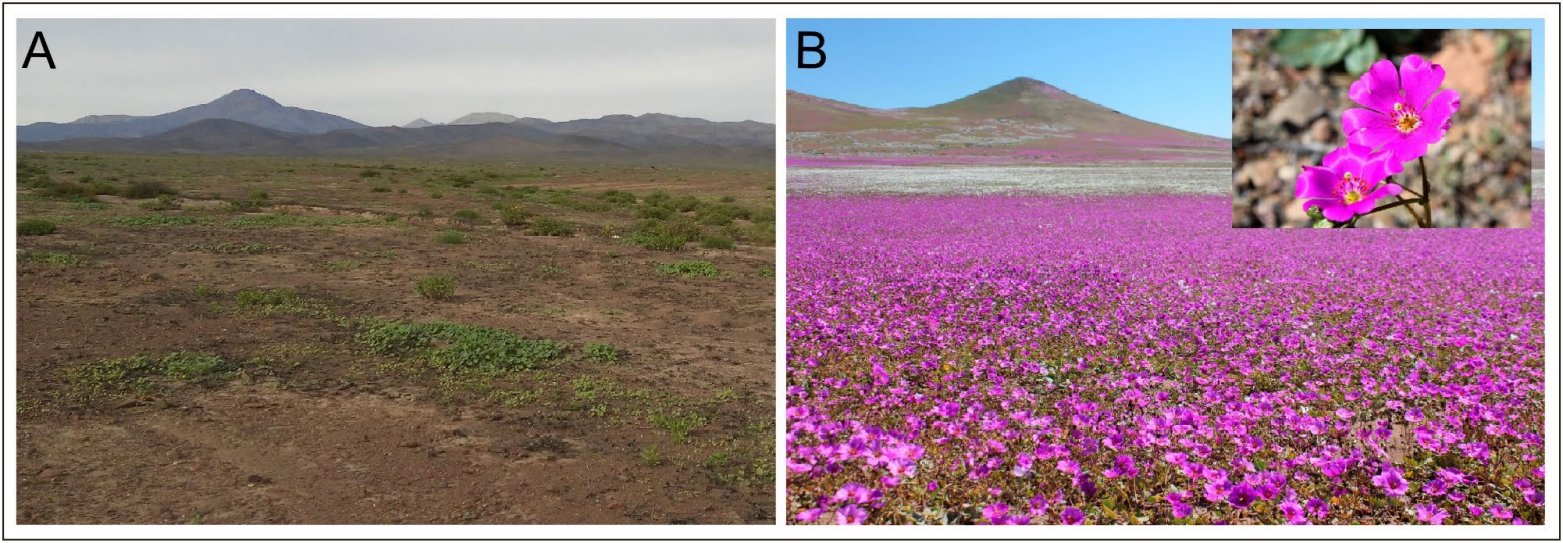
Mantles of *Cistanthe longiscapa* during preflowering (**A**) and full-flowering (**B**) stages at the 2017 flowering desert event in the Atacama Desert.

Rhizosphere soil samples were collected from three locations (27° 28′ 03′′ S, 70° 50′ 22′′ W; 28° 22′ 07′′ S, 70° 49′ 07′′ W; 28° 46′ 10′′ S, 70° 57′ 53′′ W) based on the work of Chavez et al.^18^. In this region, major FD events occurred between 1981 and 2015 and precipitation was infrequent but sometimes abundant enough to trigger the germination and blooming of dormant native seeds. Rhizosphere soil samples from representative mantles of *C. longiscapa* (Barnéoud) Carolin ex M. A. Hershkovitz (*C. longiscapa*) (Fig. 1) were collected using a cleaned spade to a depth of 0‒10 cm as previously described^19^. Fifty to 100 g of rhizosphere soil was placed into sterile plastic bags, sealed, and transported on ice to the Applied Microbial Ecology Laboratory (EMALAB) at La Frontera University, Temuco, La Araucanía, Chile.

### Isolation of culturable rhizobacterial strains

Culturable rhizobacteria from samples were obtained by platting serial dilutions of samples on different culture media (such Luria‒Bertani (LB), NM-1 minimal medium and tryptone soya agar (TSA) medium) and then by streaking the purified isolates on LB agar^20^. Briefly, 10 g of rhizosphere sample was added to a glass beaker containing 100 mL of sterile saline solution (0.8% NaCl). The suspension was vigorously shaken for 30 min at room temperature and then serial dilutions were prepared in saline solution and plated on LB agar plates. The plates were incubated for 4 days at 30 °C. A total of 72 colonies with different phenotypes (color, elevation, edges, etc.) were isolated and purified by streaking onto LB agar plates. The purified isolated strains were stored at − 80 °C in 7:3 LB:glycerol until further analysis.

### Determination of plant growth promoting traits in rhizobacterial strains

The plant growth promoting traits of the isolates were evaluated in vitro as follows. Phosphate solubilization (PS) was selected because Chilean soils have a great phosphorus retention capacity^21^. PS activity was determined on agar plates using the National Botanical Research Institute’s phosphate growth medium (NBRIP; 10 g L^−1^ d-glucose, 5 g L^−1^ Ca-phosphate, 5 g L^−1^ MgCl_2_ × 6 H_2_O, 0.25 g L^−1^ MgSO_4_ × 7 H_2_O, 0.2 g L^−1^ KCl, 0.1 g L^−1^ (NH_4_)_2_SO_4_ and 15 g agar^22^. The isolated strains were grown for 48 h at 30 °C, and clear zones surrounding colonies were measured and used as an indicator of PS activity. *Azospirillum/Herbaspirillum*-like rhizobacteria (N_2_-fixing; NF) among isolated strains were screened by using NFb (nitrogen free broth) semi-solid culture medium, with malate as the sole carbon source, as previously described^23^. The putative N_2_-fixing rhizobacteria were revealed by thin white growth near the top of the tubes. Inocula from these white zones were serially diluted in sterile 0.8% NaCl and plated onto Congo Red and Ashby’s agar media for N_2_-fixing bacteria as previously described^24^. Then, inocula from these white zones were collected, serially diluted (from 10^−1^ to 10^−5^ in sterile 0.8% NaCl) and plated on red Congo agar and Ashby agar for N_2_-fixing bacteria selection as selective culture media, where only N_2_-fixing bacteria are able to grow^24^.

The activity of 1-aminocyclopropane-1-carboxylate deaminase (ACCD), which is related to the ability to reduce ethylene synthesis, was a feature selected since ethylene is produced by plants in response to damage or environmental stress^25^. ACCD in isolated strains was determined by measuring the amount of α-ketobutyrate produced when ACCD cleaves the substrate ACC^26^. The amount of α-ketobutyrate produced (μmol) in each sample was determined by measuring the absorbance at 540 nm using a MultiskanTM GO spectrophotometer (Thermo Fisher Scientific, Waltham, MA, USA). The values obtained at an absorbance of 540 nm were compared to those of a standard curve produced using pure α-ketobutyrate at concentrations ranging from 0.0 to 1.0 mmol L^‒1^. The ACCD activity is expressed as μmol α-ketobutyrate h^‒1^ g protein. The production of tryptophan-induced auxins was determined and measured using the Salkowski colorimetric method according to the standard protocol^27^, where aliquots of bacterial culture were initially grown in DF salt minimal medium and transferred to DF salt minimal medium supplemented with 5 mM L^−1^ tryptophan. After incubation (36 h at 30 °C), bacterial cells were removed from the cultures by centrifugation (3000×*g*) and the supernatants were mixed vigorously with Salkowski’s reagent (150 mL of concentrated H_2_SO_4_, 250 mL of distilled H_2_O, 7.5 mL of 0.5 M FeCl_3_ × 6H_2_O). The auxin content, primarily indole acetic acid (IAA), was determined by measuring absorbance at 280 nm and by comparison to a standard curve produced using IAA at concentrations ranging from 0.0 to 50 μg mL^−1^. The exopolysaccharide (EPS) content, which is responsible for biofilm formation, were analyzed using the acid hydrolysis method^28^. One milliliter aliquots of 1% (w vol^−1^) cold tryptophan were added to tubes, and the samples were heated in a boiling water bath for 20 min. After cooling, the amount of EPS produced was determined at an absorbance of 500 nm by comparing the obtained values to a standard curve of glucose equivalents elaborated with pure sucrose at concentrations ranging from 0.0 to 5.0 mg mL^−1^^29^.

### Compatibility of mixed cultures

The compatibility of the isolates was examined to assemble the rhizobacterial consortia. Of the initial 72 isolates, only 23 showed plant growth promoting (PGP) traits and were subjected to the compatibility test. Strains were genotyped based on partial sequencing of 16S rRNA genes using the universal primer set 27f (5′-AGA GTT TGA TCC TGG CTC AG-3′) and 1492r (5′-TAC GGY TAC CTT GTT ACG ACT T-3′)^30^, and their growth compatibility was assayed on agar plates^31^. Briefly, 50 μL aliquots of a cell suspension of each isolate were plated onto LB agar plates and incubated for 24 h at 30 °C. After incubation, 5 μL of each of the 23 isolates was deposited onto previously inoculated plates and re-incubated for 24 h at 30 °C. Strain incompatibility was defined by the visual presence of inhibition of growth, and isolates showing incompatibility or growth inhibition capability were discarded and not used for the formulation of consortia. After testing, only 12 isolates were able to grow without inhibiting the growth of other bacteria, thus eliminating most of the *Pseudomonas* strains, and then the strains identified as the same were eliminated. Finally, only 9 isolates were selected to formulate the consortia, and they were considered different, regardless of their taxonomic affiliation, and thus presented different phenotypes (that is, expressing different PGP traits).

### Assembly of rhizobacterial consortia

Based on the results from PGP traits, genotyping, and compatibility tests, three rhizobacterial consortia were formulated using isolated strains belonging to *Bacillus*, *Paenibacillus* and *Brevibacillus* (phylum Firmicutes). Each selected strain was grown separately in LB broth to a concentration of 10^6^ CFU mL^−1^. Later, equal quantities of each strain were obtained to assemble the respective consortia. Each consortium was held at room temperature for 12 h before being applied. All three consortia were formulated to have more than three PGP traits (Table 1). Consortium “A” was composed of three strains relative to *Bacillus subtilis* and showed 1 to 3 PGP traits, whereas Consortium “B” was formulated with *B. subtilis, Paenibacillus polymyxa* and *Bacillus mojavensis* and showed three to five PGP traits. Consortium “C” consisted of *B. subtilis*, *Bacillus altitudinis* and *Brevibacillus laterosporus* and showed only one PGP trait per isolate. This last consortium did not include isolated strains with exopolysaccharide production traits.

**Table 1:** Characterization of rhizobacterial isolates from *Cistanthe longiscapa* used in the formulation of consortia.

Consortium	Isolate	Closest relatives or cloned sequences (accession no.)^†^	Plant growth-promoting traits				
			PS	NF	AU	ACC	EPS
A	11	*Bacillus subtilis* BCRC 10255 (NR_116017)	−	+	−	+	+
	13	*Bacillus subtilis* BCRC 10255 (NR_116017)	+	−	−	−	+
	14	*Bacillus subtilis* BCRC 10255 (NR_116017)	−	−	+	−	−
B	17	*Bacillus subtilis* BCRC 10255 (NR_116017)	+	+	−	−	+
	3	*Paenibacillus polymyxa* DSM 36 (NR_ 117725)	+	+	+	+	+
	15	*Bacillus mojavensis* ifo 15718 (NR_118290)	+	+	+	+	+
C	14	*Bacillus subtilis* BCRC 10255 (NR_116017)	−	+	−	−	−
	4	*Bacillus altitudinis* BCZ2 (MF954002)	+	−	+	−	−
	12	*Brevibacillus laterosporus* DSM 25 (NR_112212)	−	−	−	+	−

*PS* phosphate solubilization, *NF* growth in N-free culture médium, *AU* production of tryptophan-induced auxins, *ACCD* 1-aminocyclopropane-1-carboxylate deaminase activity, *EPS* production of exopolysaccharides.

^†^Based on partial sequencing of 16S rRNA gene and comparison with those present in GenBank by using BLASTn algorithm.

### Determination of plant growth promoting traits in rhizobacterial consortia

While the PGP traits of individual strains were initially evaluated as described above, we sought to determine whether these same traits were maintained when the strains were mixed together to form consortia. The PGP traits were measured as described above except for the production of tryptophan-induced auxins (AU), which was evaluated by high-performance liquid chromatography (HPLC) according to the methods described in the literature^32^. Briefly, overnight bacterial cultures (10 mL) were centrifuged at 3000×*g* and the supernatants were filtered through 0.22 μm membranes to remove residual discarded bacterial cells. Supernatants were analyzed by HPLC using a DAD Shimadzu, LC20AT pump, CTO 20AC furnace, DAD SPD M20A detector, and a C18 reversed-phase column (5 μm, 4.6 × 100 mm^‒2^). The mobile phase consisted of acetic acid (1.1%): acetonitrile (70:30) with a flow of 1 mL min^‒1^. The eluates were detected at 280 nm, and auxins were identified and quantified by integration of the areas under the peaks using a standard curve prepared with pure IAA that was prepared using concentrations ranging from 0 to 75 μg mL^−1^.

### Plant inoculation assay with formulated rhizobacterial consortia

Plant growth promotion by the formulated consortia was evaluated in inoculation assays performed under greenhouse conditions using the standardized “Fundo El Vergel” (Angol City, Chile) protocol with tomato (*Lycopersicon esculentum* L.) as a plant model. Commercial tomato seeds (Cal Ace variety; Seminis Vegetable Seeds, Inc.) were purchased, and assays were carried out according to the guidelines and regulations of the Agriculture Ministry for Chilean Horticulture.

**Figure 2:**
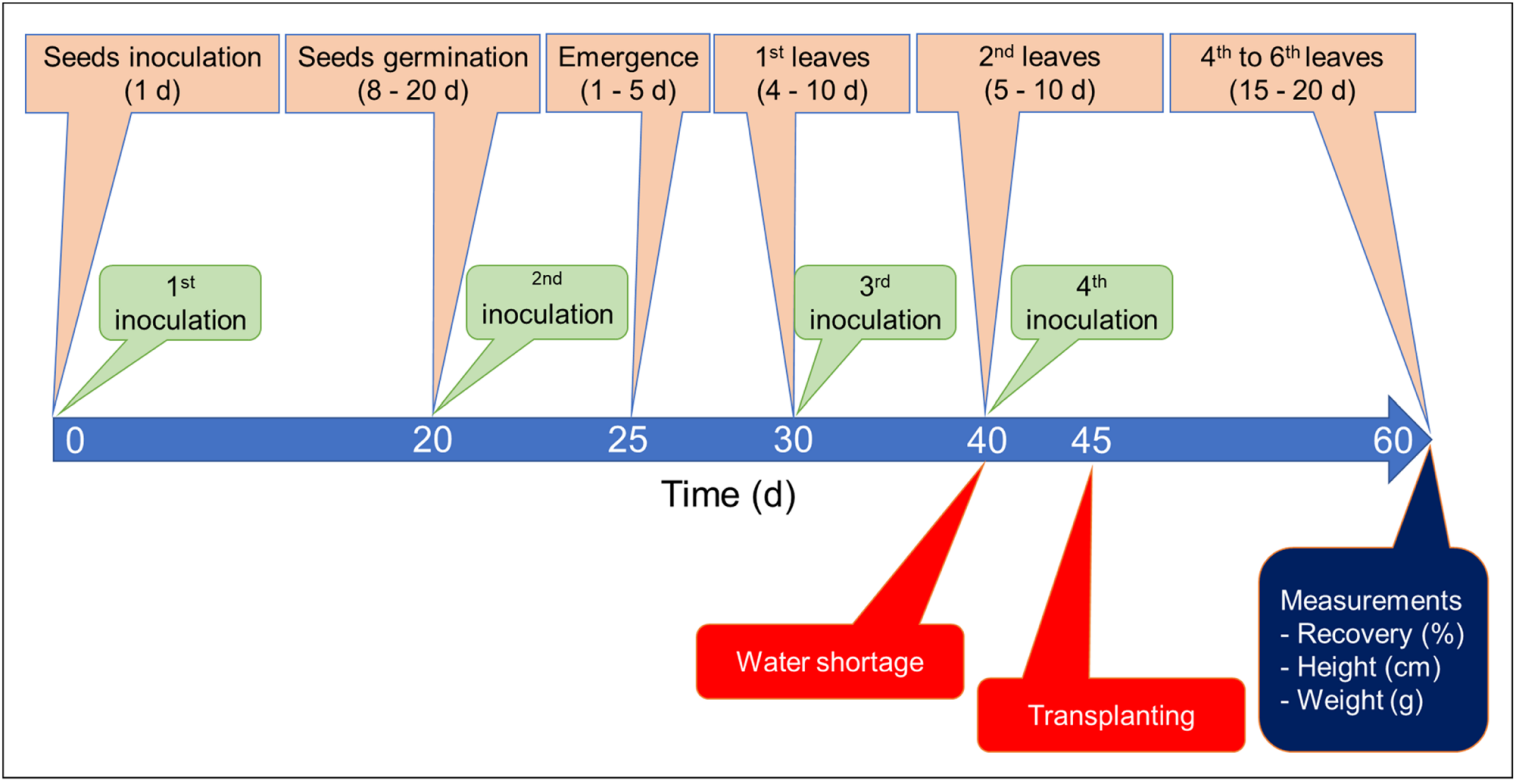
Experimental design of the plant inoculation assay with the formulated rhizobacterial consortia.

The experimental design is shown in Fig. 2. Germination of tomato seeds was evaluated after inoculation at the pre‒ and postsowing stages on day 1. This procedure was named the 1st inoculation. For the presowing treatment, 100 seeds were submerged for 24 h in 20 mL of rhizobacterial suspension containing equal concentrations of each isolate at a total cell density of 10^6^ CFU mL^−1^. For the postsowing treatment, 100 seeds were deposited into the substrate and then inoculated with 0.2 mL of inoculum per seed (10^6^ CFU mL^−1^). These inoculated and non-inoculated control seeds were sown in pots using a commercial peat-based (90% vol vol^−1^ porosity and 75 kg m^−3^ density) substrate and irrigated to 80% to 85% field capacity^33^ as the water holding capacity until germination (from 8 to 20 days). Then, the percentage germination of seeds by each consortium was evaluated^34,35^. The number of days between sowing and germination (germination time) was recorded for each treatment, and the primary root length was measured from randomly selected plants (n = 10).

Then, reinoculations were performed according to the phenological stage of tomato seedlings. A 2nd inoculation with 2 mL of the rhizobacterial suspension at 10^6^ CFU mL^−1^ was immediately applied to the germinated seeds (n = 50), and a 3rd inoculation was performed approximately at day 30 (10^6^ CFU mL^−1^) when the seedlings (n = 50) showed the first appearance of leaves. Around day 40, a 4th inoculation was performed, with the seedlings (n = 50) subjected to water scarcity stress (not irrigated) for 24, 72 and 120 h. The periods of water deficit were determined considering that the minimum and maximum number of hours that a tomato seedling can survive without irrigation before definitive transplanting, considering that maximum water consumption in tomato plants occurs during blooming and setting^36^.

Finally, 50 seedlings from each treatment were taken when two leaves were developed (approximately day 45) for transplantation into commercial coconut fiber and peat-based substrate (90% vol vol^−1^ porosity and 75 kg m^−3^ density). After transplantation, plantlets were grown for 15 days and irrigated to 80% to 85% of field capacity. The percentage of plantlets (n = 50) that recovered after water stress as well as the plant height (cm) and fresh shoot weight (g) were measured at day 60 as previously described^37^. The number of leaves developed by plants was measured after 20, 40 and 60 days according to the phenological stage of the tomato plants.

For the statistical analysis, the data obtained were subjected to a one-way analysis of variance (ANOVA) and the means were compared by Tukey’s test for multiple comparisons. Differences between treatments were considered significant at *P* ≤ 0.05.

## Results

### Assembly and determination of the PGP traits of the rhizobacterial consortia

**Figure 3:**
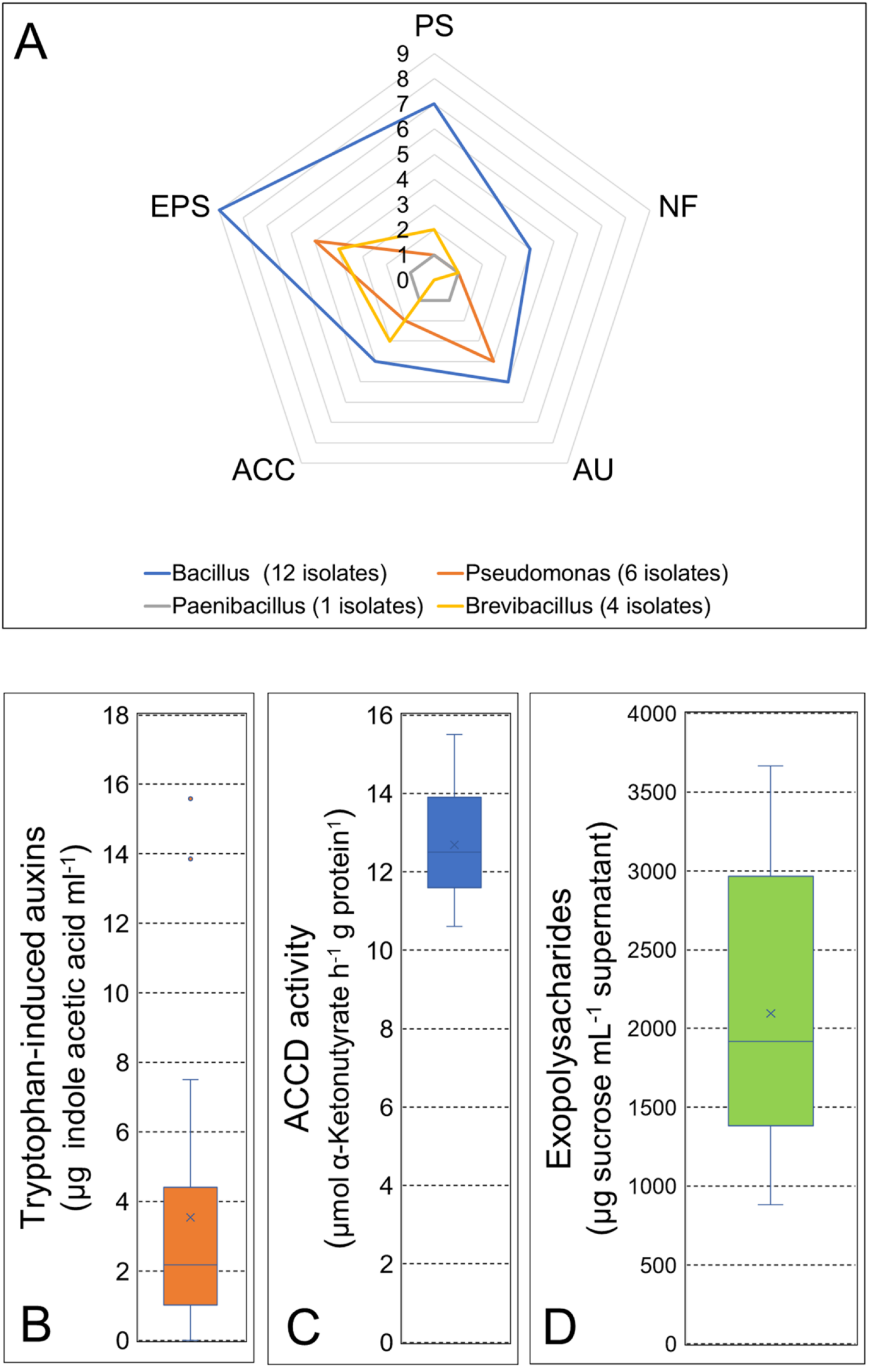
(**A**) Radial diagram showing the number of plant growth-promoting traits found in rhizobacterial strains isolated from *Cistanthe longiscapa*. *PS* phosphate solubilization, *NF* growth in N-free culture médium, *AU* production of tryptophan‒induced auxins (**B**); *ACCD* 1-aminocyclopropane-1-carboxylate deaminase activity (**C**); and *EPS* exopolysaccharide production (**D**).

Analyses of the partial sequences of 16S rRNA genes indicated that selected isolated strains belonged to the genera *Bacillus* (12), *Pseudomonas* (6), *Brevibacillus* (4) and *Paenibacillus* (1). The *Bacillus* isolates had the greatest number of PGP traits, particularly the production of EPS and P solubilization (Fig. 3A). The production of tryptophan-induced auxins by selected isolated strains ranged from 0.1 to 15.6 μg of indole acetic acid mL^−1^ (Fig. 3B), the activity of ACCD ranged from 10.6 to 15.1 μmol of α-ketobutyrate h^−1^ g protein^−1^ (Fig. 3C), and the production of EPS ranged from 883 to 3667 μg of sucrose equivalents mL^−1^ of supernatant (Fig. 3D). When the compatibility of isolated strains was tested on agar plates, strains belonging to the genus *Pseudomonas* provoked growth inhibition of the other assayed strains, including *Pseudomonas* itself. Consequently, a decision was made to use only representatives of the Firmicutes taxa for consortium assembly (Table 1). Initial mixture analyses indicated that the assembled consortia not only maintained their PGP traits but that some had higher PGP activities, with values of tryptophan-induced auxin production of indole acetic acid mL^−1^ increasing from 118.2 to 122.6 μg (3.7% increase), ACCD activity increasing from 27.1 to 68.7 μmol of α-ketobutyrate h^−1^ g protein^−1^ (253.5% increase), and EPS production increasing from 1085.3 to 3077.5 μg sucrose mL^−1^ supernatant (283.6% increase) (Table 2). Notably, Consortium A, which was formulated with only *B. subtilis* strains, showed significantly (*P* ≤ 0.05) higher values of PGP traits, and Consortium C showed an EPS production trait that was not observed when the individual strains were characterized.

**Table 2:** Plant growth-promoting traits in rhizobacterial consortia from *Cistanthe longiscapa*.

Consortium	Plant growth-promoting traits			
	PS (cm^†^)	AU (μg IAA mL^‒1^)	ACCD (µmol α-KB mg^‒1^ protein)	EPS (µg sucrose mL^‒1^ supernatant)
A	1.80 ± 0.08* b^‡^	122.6 ± 8.98 a	68.7 ± 2.5 a	3077.5 ± 314.2 a
B	1.80 ± 0.07 b	118.2 ± 7.47 b	45.8 ± 3.9 b	3072.8 ± 768.1 a
C	1.95 ± 0.05a	118.4 ± 6.46 b	27.1 ± 5.0 c	1085.3 ± 264.4 b

*PS* phosphate solubilization, *AU* production of tryptophan-induced auxins, *ACCD* 1-aminocyclopropane-1-carboxylate deaminase activity, *EPS* exopolysaccharides production, *IAA* indole acetic acid, *KB* α-ketobuty rate.

*Values represent the means ± standard deviation of *n* = 3.

^†^Ratio is calculated as clear halo diameter/colony diameter of phosphate solubilization.

^‡^Different letter in each column denote significant differences (*P* ≤ 0.05) by ANOVA followed by Tukey’s post-hoc test.

### Plant inoculation assay with formulated rhizobacterial consortia

The impacts of the formulated consortia on the germination percent and germination time are presented in Table 3. Prior to sowing, the seeds inoculated with Consortium B showed a significantly (*P* ≤ 0.05) greater germination percentage (97%) compared with the uninoculated controls (90%) and consortia A and C (90% and 92%, respectively). However, inoculation with Consortium B also resulted in a significantly greater (P ≤ 0.05) germination time (12 days) relative to that of seeds in the control (18 days) and other consortia (9 and 10 days) treatments. When inoculated at sowing, the seeds inoculated with Consortia B and C showed significantly (*P* ≤ 0.05) higher germination rates (91% and 92%, respectively) compared with the control (86%) and significantly (*P* ≤ 0.05) shorter germination times (12, 15 and 10 days for consortia A, B and C, respectively) compared with the control (18 days).

**Table 3:** Percentage and time of germination of tomato seeds inoculated with rhizobacterial consortia from *C. longiscapa* at presowing and sowing.

Inoculation time	Consortium	Germination (%)	Germination time (days)
Pre-sowing	Control	90 ± 5* b^†^	8 ± 5 b
	A	90 ± 5 b	9 ± 5 b
	B	97 ± 1 a	12 ± 3 a
	C	92 ± 2 b	10 ± 3 a
Sowing	Control	86 ± 8 b	18 ± 2 a
	A	83 ± 8 b	12 ± 3 b
	B	91 ± 2 a	15 ± 4 b
	C	92 ± 2 a	10 ± 3 b

*Values represent the means ± standard deviation of 50 seeds per treatment.

^†^Different letter in each column denote significant differences (*P* ≤ 0.05) by ANOVA followed by Tukey’s post-hoc test.

Primary root length was measured after 15 days for the seeds the consortia A, B and C and control treatments prior to sowing or at sowing. The results in Table 4 show that at presowing, the seeds inoculated with the consortia showed shorter primary root lengths (3.1 to 3.5 cm) than the control treatment (5.5 to 5.6 cm). In contrast, at sowing, the seeds inoculated with Consortium B had the longest primary roots (7.6 to 8.1 cm) while the seeds inoculated with Consortium C had similar primary root lengths in both the presowing and sowing treatments.

**Table 4:** Primary root lengths of 15-day-old tomato seedlings inoculates with formulated rhizobacterial consortia.

Inoculation time	Consortium	Primary root lengths (cm)
Pre-sowing	Control	5.5 ± 0.5* b^†^
	A	3.1 ± 0.5 c
	B	7.6 ± 0.2 a
	C	6.8 ± 0.2 a
Sowing	Control	5.6 ± 0.3 c
	A	3.8 ± 0.2 d
	B	8.1 ± 0.3 a
	C	6.9 ± 0.2 b

*Values represent the means ± standard deviation of 10 seeds per treatment.

^†^Different letter in each column denote significant differences (*P* ≤ 0.05) by ANOVA followed by Tukey’s post-hoc test.

The impact of no irrigation on the recovery and growth of plantlets and the number of leaves developed by seedlings was also evaluated, and the results are summarized in Table 5. All the inoculated plants (100%) survived 24 h without irrigation, while the uninoculated control plants only showed 92% recovery. There was a dramatic and significant (*P* ≤ 0.05) effect of inoculation on plant recovery after 72 h without irrigation, and the plants inoculated with Consortium A had 100% recovery compared with the controls (67% recovery). Moreover, even after 120 h of water shortage, the inoculated plants showed significantly (*P* ≤ 0.05) greater recovery (from 88 to 96%) than the control (54%).

**Table 5:** Recovery and growth of tomato plants inoculated with rhizobacterial consortia and exposed to water shortage stress at different time intervals (24, 72 and 120 h) prior to transplant to definitive substrate.

Water shortage	Consortium	Recovery (%)	Height (cm)*	Weight (g)*	Number of leaves (days after transplant)		
					20 days	40 days	60 days
24 h	Control	92 ± 5** b^†^	51.3 ± 5.3 b	197.7 ± 8.9 b	4.1 ± 0.2 b	5.1 ± 0.1 d	6.8 ± 0.2 a
	A	100 ± 1 a	59.4 ± 4.2 a	200.4 ± 9.8 b	4.9 ± 0.1 a	5.6 ± 0.1 c	6.1 ± 0.3 b
	B	100 ± 1 a	58.7 ± 4.1 a	189.8 ± 8.6 b	4.0 ± 0.2 c	6.1 ± 0.2 b	6.9 ± 0.2 a
	C	100 ± 1 a	54.8 ± 3.5 b	214.7 ± 7.3 a	3.5 ± 0.3 d	6.5 ± 0.1 a	7.1 ± 0.1 a
72 h	Control	67 ± 10 c	41.5 ± 3.7 b	178.7 ± 9.8 c	3.2 ± 0.1 a	5.1 ± 0.3 b	5.9 ± 0.1 c
	A	100 ± 1a	58.8 ± 4.3 a	198.5 ± 4.5 a	3.9 ± 0.2 a	5.4 ± 0.2 b	6.5 ± 0.1 b
	B	92 ± 5 b	59.5 ± 5.2 a	187.3 ± 6.2 b	2.9 ± 0.1 c	5.9 ± 0.2 a	6.8 ± 0.1 a
	C	92 ± 5 b	60.1 ± 3.7 a	195.8 ± 5.7 a	4.2 ± 0.1 a	6.1 ± 0.1 a	6.5 ± 0.2 b
120 h	Control	54 ± 10 c	37.3 ± 5.3 c	146.9 ± 8.3 c	3.0 ± 0.2 a	4.5 ± 0.1 c	4.7 ± 0.1 c
	A	96 ± 2 a	48.5 ± 3.2 b	167.5 ± 6.5 b	2.7 ± 0.2 b	4.9 ± 0.2 a	5.7 ± 0.2 b
	B	88 ± 5 b	51.3 ± 4.9 b	171.1 ± 7.2 a	2.5 ± 0.3 b	4.8 ± 0.3 b	5.8 ± 0.1 b
	C	92 ± 3 b	52.8 ± 2.2 a	175.3 ± 6.3 a	3.1 ± 0.2 a	5.3 ± 0.2 a	6.3 ± 0.1 a

*Growth was measured after 60 days since transplant.

**Values are means ± standard deviation of 50 seedlings per treatment.

^†^Different letter in the column denote significant differences (*P* ≤ 0.05) by ANOVA followed by Tukey’s post-hoc test.

The beneficial effect of the consortia on tomato plants was also evidenced 15 days after emergence, particularly in seedlings subjected to water shortage treatments for 72 h and 120 h (Table 5). Inoculated plants exposed to 24 h of water shortage showed higher heights (averaging 54.8 to 59.4 cm) than the uninoculated controls (average 51.3 cm). Similar effects were obtained for fresh shoot weight, with significantly greater weights were seen in seedlings inoculated with Consortium A and C (200.4 g and 214.7 g, respectively) compared with the uninoculated controls (197.7 g). At 72 h of water shortage, the three consortia resulted in plants with heights close to 60 cm, which was statistically higher (*P* ≤ 0.05) than the control (41.5 cm). Significantly greater weights (*P* ≤ 0.05) were also obtained in plants inoculated with Consortium A (198.5 g) and C (195.8 g) compared with control plants (178.7 g). A similar trend was observed at 120 h of water shortage, where plants had significantly (*P* ≤ 0.05) greater heights (from 48.5 to 52.8 cm) and weights (from 167.5 to 175.3 g) in plants inoculated with rhizobacterial consortia compared with those without inoculation (37.9 cm and 146.9 g). Generally, plants inoculated with Consortium A had better recovery from water stress, whereas plants inoculated with Consortium C showed better growth.

The number of leaves formed after transplantation was used as an indicator of the impact of water stress (Table 5). Seedlings receiving 24 h of water stress showed no great differences after inoculation with consortia A, B or C compared with the control plants 20 days after transplanting. Similarly, 60 days after transplanting, the differences in leaf number were not significant. Seedlings receiving 72 h of water shortage stress started with the same patterns as seedlings receiving 24 h of stress. Inoculation of plants with Consortium B had the smallest effect on leaf development after 20 days of recovery. After 60 days, however, the results seen with plants receiving Consortium B were similar to those receiving consortia A and C and the control treatment. The 120-h water stress treatment similarly affected the leaf numbers on all plants after 20, 40 and 60 days of recovery. However, the effect of inoculation on leaf number was significantly greater than that seen with the control (more than 6 to 7 leaves developed in 60 days versus 4 to 5 leaves in the control). In addition, inoculation with Consortium C led to greater leaf development in the same period. Inoculation with consortia A and B had a positive effect on leaves, although the effect was less than that observed with Consortium C.

## Discussion

Our study revealed the occurrence of several bacterial strains with PGP traits in the rhizosphere of *C. longiscapa* during an FD event in the Atacama Desert, for which few subjects have been studied thus far. The strains were taxonomically affiliated with members of the genera *Bacillus*, *Paenibacillus*, *Brevibacillus*, and *Pseudomonas*. Diverse studies have revealed the presence of potential PGPR associated with plants in arid ecosystems, including the Atacama Desert^13,20^. Both *Bacillus* and *Pseudomonas* spp. strains showing growth-promoting traits are commonly used as soil inoculants and recognized by their resilience to harsh desert conditions^38,39^. Similarly, members of *Paenibacillus* and *Brevibacillus* have also been found in the rhizosphere of desert plants and proposed for use as PGPR^40^.

The EPS production was the main PGPR trait found in isolated strains. EPSs have long been recognized for the important benefits they provide to microbiota, either as single organisms, in binary associations, or in heterogeneous mixed communities in adverse environments^41^. It has been proposed that EPSs function as protect microorganisms from ultraviolet radiation, extreme temperature, extreme pH, high salinity, high pressure, and poor nutrients, among others harsh features found in extreme environments^42^. EPSs have also been shown to protect plant-associated rhizobacteria from water stress by enhancing water retention and by enhancing root colonization and attachment by the formation of a network of fibrillar material that permanently connects the cells to the root surface^43^. It should be noted that our isolated strains produced greater amounts of EPS than *Bacillus* and *Paenibacillus* isolates found among rhizobacteria from desert plants^44^. In addition, the *Bacillus*-enhanced EPS production capacity has been used to increase soil moisture for maize grown under drought stress^45^.

Rhizobacterial *Bacillus* strains with auxin production and ACCD deaminase activity have also been isolated from plants growing in arid environments^46,47^. ACCD production by our strains was much higher (10.6 to 15.1 μmol of α-ketobutyrate h^−1^ g protein^−1^) than that reported in 2019 in rhizobacteria from native plants grown in the Andean altiplano^48^ of the Atacama Desert (0.8 to 3.3 μmol of α-ketobutyrate h^−1^ g protein^−1^). However, these amounts were lower than those of other strains (4.6 from *B. licheniformis* K11 to 402.1 from *Enterobacter cloacae*) isolated from plants under drought conditions^49,50^. The quantity of tryptophan‒induced auxins produced by our isolated strains was lower (from 0.1 to 15.6 μg mL^−1^) than those observed in other rhizobacteria than *Bacillus* in desert soils but similar to those in the same environment^51^ (from 23 to 37 for non-*Bacillus* bacteria, and 4.2 to 9.2 g mL^−1^ for *Bacillus* species).

We also showed that some strains were compatible, which made it possible to assemble a consortia of PGPR. Based on the results from compatibility tests, our consortia were only formulated using members of the phylum Firmicutes. While individual strains have been reported to be PGPR, we expected synergistic or enhanced activity in each formulated consortium^52^. This idea was confirmed in our study, where all assayed consortia not only maintained their individual PGP traits (e.g., phosphate solubilization) but also had enhanced ACCD activity compared with that obtained for individual isolated strains. In addition, while there was a trend toward greater production of tryptophan-induced auxins in the consortia compared with individual cells, we cannot directly compare the results because different methods were used to quantify auxins in individual cells (Salkowsky reagent) vs consortia (HPLC). Interestingly, Consortium C, which was formulated with isolated strains that individually did not show EPS production, showed EPS production when the strains were mixed together. This finding was likely due to the general synergistic effects that allow members of Consortium C to optimize the use of resources^45^. In addition, a protective effect against water stress has also been observed in maize, pea and vetch plants treated with EPS-producing rhizobacteria^53–55^. It is known that EPSs provide a microenvironment that holds water and dries more slowly compared with the surrounding environment, thus protecting bacteria and roots of agriculturally relevant vegetables against desiccation^56^.

In relation to the tomato inoculation assay with the formulated rhizobacterial consortia, the application of consortium at sowing and presowing resulted in a significantly (*P* ≤ 0.05) higher germination percentages and less time required for seed germination in seeds inoculated at sowing compared with the uninoculated controls. Since germination is mediated by ethylene production^57^ and Consortium A has high ACCD activity, the reduced germination percentage and the number of days to germination would be associated with this feature. This was particularly evident in seeds inoculated with Consortium B and Consortium C. The addition of PGPR strains has been previously shown to increase seed germination and facilitate the production of healthy groundnut and wheat seedlings^31,58^. Inoculated seeds at presowing took more days to germinate than did the control treatment. Despite this, inoculation of tomato with any of our consortia resulted in greater germination percentages, especially for Consortium B. Considering that Consortium A had the greatest production of AU, which is related to seed germination, at least as shown for soybean plants^59^, the greater production of this phytohormone could repress seed germination, as observed with consortia B and C, which had a significantly higher germination percentage compared with the plants in the Consortium A and control treatments.

When tomato seedlings were subjected to different periods of water shortage stress, our results revealed a significantly (*P* ≤ 0.05) higher percentage of recovery in seedlings inoculated with the rhizobacterial consortia compared with uninoculated controls. Similarly, although a significant effect of rhizobacteria consortia on plant growth was not observed in seedlings exposed to 24 h of water shortage stress, inoculation with the three formulated consortia resulted in significantly greater plant growth when longer periods of no irrigation were applied (72 h and 120 h). Some studies have indicated that physiological changes in a wide variety of plants, such as maize, wheat, soybean^60^ and lavender, induced by microorganisms result in enhanced tolerance to drought stresses by PGP mechanisms, including EPS production, auxin production and ACCD activity. ACCD activity is recognized as a main PGP mechanism to ameliorate abiotic stress in plants^61^, and numerous studies have demonstrated that ACCD-producing rhizobacteria can increase the fresh and dry weights of tomato and pepper seedlings and reduce ethylene production under drought stress^62^. Since transplanting shock results in tomato plant growth retardation and developmental delay and sometimes seedling death^63^, the application of a PGP consortium would help to ameliorate this negative effect. Furthermore, in lettuce nurseries using bacterial biostimulants, transplant shock was reduced compared to a control treatment^64^. In addition, the primary root length after germination was used as an indicator of a plant’s growth capacity in the substrate and ability to reach nutrients and water from soil. As shown by our results, excessive AU production can explain the shorter primary roots measured in plants treated with Consortium A, which was also previously observed in soybean plants^59^. In contrast, consortia B and C showed similar AU production but lower AU production than Consortium A. Interestingly, the primary root length of plants under the influence of consortia B and C showed longer primary roots. These results implied better establishment and further seedling shoot growth and development of tomato plants.

As the main photosynthetic organs, leaves are important for the optimum growth and development of plants. In tomato production, the endurance of plantlets is strictly related to the number and turgor of leaves. As shown by our results, plants subjected to the consortium effect developed more leaves under more days of water shortage stress. The cumulative effect of PGP traits from the consortium would explain these observations. The main production of ACCD, especially with Consortium A, resulted in the positive development of plants under water shortage stress^65^. Seedlings commonly experience temporary periods of abiotic stresses, resulting in necessary root and shoot metabolic and structural adjustments to withstand stress conditions. Inoculation of seedlings with PGPR can improve the recovery and development of vegetables (e.g., tomato and lettuce) after exposer to stress, such as water scarcity, drought, high salt content and others^66^. In our results, consortia B and C showed the most promising results in the recovery and growth of tomato seedlings compared to the control.

Therefore, the formulation of PGPR consortia that provide a higher tolerance of vegetables to water scarcity may be pivotal as a complementary technology to face the current scenario of climate change in the Mediterranean and semiarid and arid agricultural zones worldwide^63,67,68^, including northern Chile, where the availability and quality of water are critical to support vegetable production for human consumption.

## Conclusions

This current study revealed the occurrence of culturable rhizobacteria (mostly *Bacillus* and *Pseudomonas*) harboring PGP traits in the rhizosphere of *C. longiscapa* growing during an FD event. Moreover, based on their taxonomic affiliation, compatibility tests and PGP traits, we successfully formulated three rhizobacterial consortia that improved the seed germination, recovery and growth of tomato seedlings exposed to water shortage stress under commercial greenhouse conditions. In this sense, desert plants are an important source of plant growth-promoting rhizobacteria (PGPR) with all the traits for thriving in extreme conditions. Therefore, the application of isolated native rhizobacteria adapted to arid conditions could be a valuable tool to improve the growth and tolerance of vegetables under water scarcity as a result of drier and harsher environments under climate change scenarios, which is especially true for the production of seedlings that are under pronounced stress periods during transport from production to the field. Moreover, the use of PGP consortia could improve plantlet recovery to transplant shock. Further studies on the interactions between components of autochthonous soil and rhizosphere microbiota are necessary to understand the bacterial assemblages. The potential of PGPR isolated from desert plants represents a special parameter to validate their use at a commercial scale.
